# Increased Expression of LYNX1 in Ovarian Serous Cystadenocarcinoma Predicts Poor Prognosis

**DOI:** 10.1155/2020/1392674

**Published:** 2020-11-25

**Authors:** Hui Liu, Ao Wang, Yushan Ma

**Affiliations:** ^1^Department of Anesthesiology, West China Second Hospital of Sichuan University, Key Laboratory of Birth Defects and Related Diseases of Women and Children, Sichuan University, Ministry of Education, Chengdu, China; ^2^Department of Gynecology and Obstetrics, West China Second Hospital of Sichuan University, Key Laboratory of Birth Defects and Related Diseases of Women and Children, Sichuan University, Ministry of Education, Chengdu, China

## Abstract

Few studies have reported the function of LYNX1 in ovarian cancer. We retrieved LYNX1 gene expression data and clinical information of 376 patients with ovarian cancer from The Cancer Genome Atlas (TCGA) project website. Wilcoxon signed-rank test and logistic regression were used to analyze the relationship between clinical pathologic features and LYNX1 expression. The Kaplan–Meier method was used to draw survival curves of patients, and Cox regression was used to calculate the relationship between LYNX1 expression and survival rate or the clinicopathological characteristics of the patients. Gene set enrichment analysis (GSEA) was performed, and the correlation between LYNX1 expression and cancer immune infiltrates was investigated via single sample gene set enrichment analysis (ssGSEA). High LYNX1 expression in ovarian serous cystadenocarcinoma (OVs) was associated with tumor residual disease (RD). In Kaplan–Meier survival analysis, patients with OVs who also displayed high LYNX1 expression had decreased overall survival (OS) and disease-specific survival (DSS) than those with low LYNX1 expression. Univariate analysis also supported that patients with high LYNX1 expression had lower OS than those with low LYNX1 expression. LYNX1 expression has the potential to be a prognostic molecular marker of poor survival in OVs.

## 1. Introduction

Ovarian cancer is a common lethal malignancy in women and is the most common cause of gynecologic cancer deaths [1]. China has a relatively low incidence rate of ovarian cancer, but the large population translates to an estimated 52,100 new cases and 22,500 related deaths in 2015 [2]. The American Cancer Society estimated that, in the year 2020 in the United States, 21,750 women would suffer from ovarian cancer and that 13,940 of them would die of this disease [1]. The most common histological subtype is ovarian serous carcinoma. Most patients with ovarian cancer are diagnosed in advanced stages because of the lack of specific symptoms and the absence of effective early diagnostic methods, which lead to a poor prognosis [3–5].

In recent years, common serum biomarkers used to monitor ovarian cancer progression and prognosis include CA125 and HE4, which are also commonly used to detect ovarian cancer recurrence after surgery or chemotherapy [6, 7]. However, these biomarkers lack both specificity and high sensitivity in predicting cancer metastasis, recurrence, and prognosis. Therefore, the development of more sensitive and specific biomarkers for the early diagnosis of patients with ovarian cancer is urgently needed.

Lynx1 (Ly6/Neurotoxin 1), the first three-fingered prototoxin found in the central nervous system of mammals, is a glycosylphosphatidylinositol- (GPI-) anchored protein that regulates nicotinic acetylcholine (Ach) receptors (nAChRs) in the brain [8]. The main function of nAChRs is to act as ligand-gated ion channels, which are responsible for regulating signal transduction at the junction of the central and peripheral nervous systems and at neuromuscular junctions [9]. Moreover, nAChRs also have been found in nonexcitable cells like immune and epithelial cells [10]. In addition to being involved in epithelial cell proliferation, differentiation, migration, and apoptosis, these nonneuronal receptors also control inflammation and regulate genes [11, 12]. It has been reported that different nAChR subunits are expressed in epithelial cell carcinomas such as lung cancer, mesothelioma, and colon cancer [13].

LYNX1 is expressed in different cell types and organs. The mRNA and protein expression of the LYNX1 has been found in lung, colon, epidermis, and breast cancer cells [14], and in lung adenocarcinoma A549 and colon carcinoma HT-29 cell lines, the colocalization of LYNX1 with *α*7-nAChRs has been reported in cell membranes [14]. In lung adenocarcinoma A549 cells, endogenous LYNX1 expression controls the nicotine-induced upregulation of *α*7-nAChRs that occurs during cell growth. However, it is unclear whether and by what mechanism LYNX1 has a potential function in lung cancer progression and tumor immunology. Recombinant water-soluble LYNX1 (ws-LYNX1) variants, which are not anchored by GPI, inhibited the growth of A549 cells, causing cell cycle arrest by modulating *α*7-nAChRs and activating different intracellular signaling pathways including p38, c-Jun N-terminal kinase, protein kinase C (PKC)/inositol trisphosphate (IP3), and mitogen-activated protein/extracellular-signal-regulated kinase pathways. After ws-LYNX1 treatment in A549 cells, the proapoptotic and anticancer protein p53 was phosphorylated as well as different kinases that are involved in regulating transcription, cell growth, adhesion, and differentiation [14].

In the current study, we used The Cancer Genome Atlas (TCGA), gene set enrichment analysis (GSEA), and Kaplan–Meier plotter to comprehensively analyze the expression of LYNX1 and its relationship with the prognosis of patients with ovarian serous cystadenocarcinoma (OVs). In addition, we used the single sample gene set enrichment analysis (ssGSEA) to investigate the correlation between LYNX1 and tumor-infiltrating immune cells. Moreover, we used the removeBatchEffect function in the limma package to perform batch effect correction on the TCGA data set [15]. Our results illustrate the significance of LYNX1 in OVs and explore the potential mechanism of LYNX1 in regulating the prognosis of OVs patients.

## 2. Materials and Methods

### 2.1. Editorial Policies and Ethical Considerations

This study does not contain any studies with human participants or animals performed by any of the authors.

### 2.2. Collection of LYNX1 Data from TCGA

Data for mRNA expression (mRNA SeqV2) and clinical information of human ovarian cancer were retrieved from TCGA project (https://tcga-data.nci.nih.gov/tcga/) [16]. The expression profile of LYNX1 was extracted from TCGA RNA-seq data of 376 OVs patients. The corresponding clinical prognosis (overall survival, OS; disease-specific survival, DSS) information was obtained from the University of California Santa Cruz (UCSC) Xena (https://xenabrowser.net/heatmap/) [17]. We converted the data related to the LYNX1 from the high-throughput sequencing fragments per kilobase per million (HTSeq-FPKM) format to the transcripts per kilobase million format, with the preservation and further analysis of the data and clinical materials of Level 3 of expression level in ovarian cancer patients closely followed. In this study, clinical data and prognostic information of all available samples were extracted, and prognostic indicators mainly included OS and DSS. Patients who died from causes other than the disease being studied are not counted in this measurement.

### 2.3. Analysis of Gene Set Enrichment

GSEA (http://software.broadinstitute.org/gsea/index.jsp) is a computational method determining whether a priori defined set of genes shows statistically significant, concordant differences between two biological states [18]. In our study, GSEA was the first to rate an ordered list of all genes according to their correlation with LYNX1 expression. GSEA was carried out to elucidate the significant survival difference observed between groups expressing high and low levels of LYNX1. Moreover, set permutations were performed 1000 times for each analysis. Expression profiles of LYNX1 were used as phenotypic labels, and we used nominal *p* values and normalized enrichment scores (NES) to rank the pathways with LYNX1 enrichment in each phenotype.

### 2.4. Immune Infiltration Analysis

The marker of 24 immune cells was extracted from the research of Bindea and colleagues [19]. The ssGSEA method was used to analyze the infiltration of 24 types of immune cells in the tumor, and Spearman's correlation was used to analyze the correlation between the LYNX1 and these 24 types of immune cells. The strength of the association between immune-infiltrating cells and LYNX1 was used in the following absolute values: 0.00–0.05, very weak; 0.06–0.10, weak; 0.11–0.15, moderate; and >0.15, strong. For statistical analyses, *p* values < 0.05 were considered statistically significant.

### 2.5. Statistical Analysis

The R software (version.3.6.1; http://www.Rproject.org) was used for all statistical analyses. Wilcoxon sign-rank test and logistic regression analysis were used to analyze the relationship between clinicopathological features and LYNX1. The uni- and multivariable regressions were performed with dichotomized LYNX1 expression scores. The Kaplan–Meier method was used to construct survival curves, and the log-rank test was used to analyze the differences between survival curves. The individual hazard ratio (HR) of the operating system was estimated by univariate Cox proportional risk regression. Elements with *p* < 0.05 in the significance variables of univariate analysis were included in multivariate Cox analysis. HR and 95% confidence intervals (CI) were measured to estimate the HR of individual factors. The *p* values of all results were bilateral, with 0.05 indicating significance.

## 3. Results

### 3.1. Patient Characteristics

Clinical data and expression data of 376 cases of OVs were downloaded from TCGA data in December 2019 (Table 1). A total of 198 patients under the age of 60 developed OVs, accounting for 52.66% of the total number of patients in this study, while 178 patients aged 60 or above accounted for 47.34%.

In this study cohort, most patients were stage III (293 cases; 78.55%), followed by stage IV (57 cases; 15.28%), and the smallest numbers were in stage I (1 case; 0.27%) and stage II diseases (22 cases; 5.9%). Primary therapy outcomes of OVs included 7.28% in stable disease (SD), 8.94% in progressive disease (PD), 14.24% in partial remission (PR), and 69.54% in complete remission (CR). The cohort was followed up for 12 years, from 2006 to 2018. Median follow-up for subjects alive at last contact was 39.7 months (range 0–182.7 months). The median follow-up for subjects alive at last contact was 41.63 months in the high LYNX1 expression group and 50.37 months in the low LYNX1 expression group. Of those patients who were followed up, 146 (38.83%) survived and 230 (61.17%) died. The largest number of these had tumors (246 cases; 74.32%), and the remaining 85 cases (25.68%) were tumor free. Three hundred and thirty-three patients were assessed for residual tumor disease, among whom 267 (80.18%) had no residual disease (NRD), while 66 (19.82%) had residual tumor disease.

### 3.2. Association with LYNX1 Clinicopathological Variables and Expression

**Figure 1:**
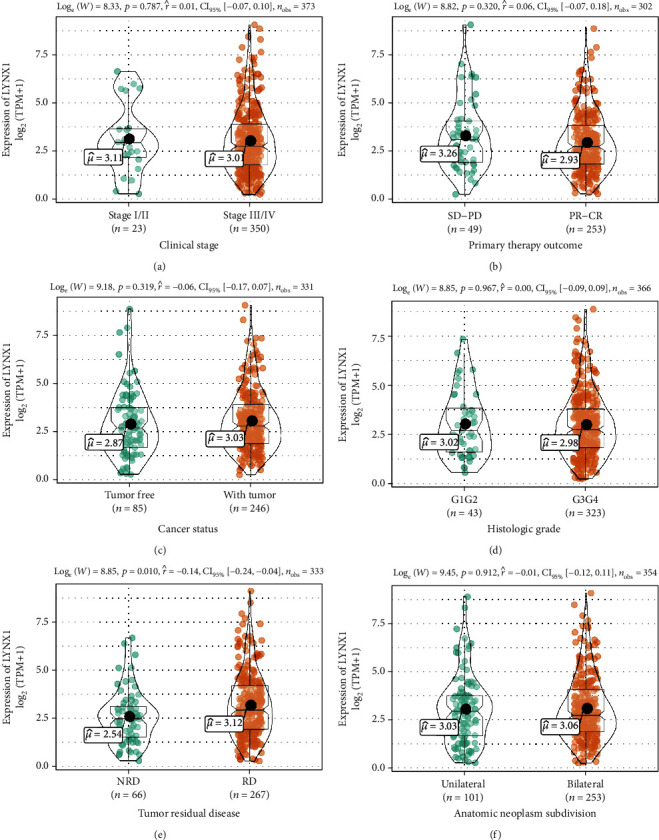
Association with LYNX1 expression and clinicopathological characteristics including (a) clinical stage, primary therapy outcome (b), cancer status (c), histologic grade (d), tumor residual disease (e), and anatomic neoplasm subdivision (f) in patients with ovarian serous cystadenocarcinoma (OVs) in The Cancer Genome Atlas (TCGA) cohort.

To better understand the relevance and underlying mechanisms of LYNX1 expression in OVs, we investigated the relationship between the LYNX1 expression and clinical characteristics of 376 OVs samples. As shown in Figure 1, increased LYNX1 expression was enriched in clinical stages III/IV (*p* = 0.787), primary therapy outcomes PR-CR (*p* = 0.320), cancer status with tumor (*p* = 0.319), histological grade G3/G4 (*p* = 0.967), tumor residual disease (RD) (*p* = 0.010), and anatomic neoplasm subdivision bilateral (*p* = 0.912).

As shown in Table 2, univariate analysis revealed that as a categorical dependent variable, LYNX1 expression was associated with poor prognostic clinicopathological characteristics via logistic regression. Increased LYNX1 expression in OVs significantly associated with tumor RD (odds ratio, OR = 2.08 for NRD vs. RD) (*p* = 0.01) and age (OR = 0.55 for <60 vs. ≥60) (*p* = 0.004).

### 3.3. Multivariate Analysis and Survival Outcomes

**Figure 2:**
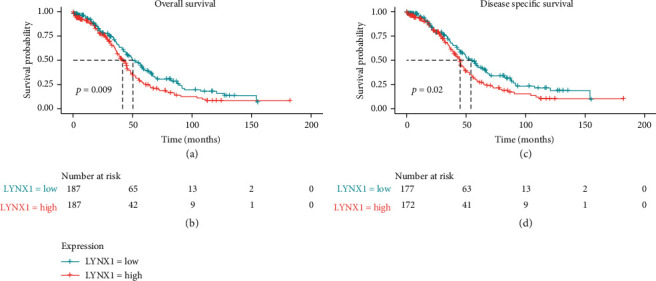
Impact of LYNX1 expression on overall survival (OS) and disease-specific survival (DSS) in ovarian serous cystadenocarcinoma (OVs) patients in The Cancer Genome Atlas (TCGA) cohort.

Kaplan–Meier survival analysis showed that OS and DSS with low amounts of LYNX1 represented a better prognosis than that with high expression of LYNX1 (*p* = 0.009). The results showed that high LYNX1 values correlated significantly with a poor OS (*p* = 0.009) and DSS (*p* = 0.02) by univariate analysis (Figure 2).

Other clinicopathological variables were associated with poor OS including primary therapy outcome, cancer status, tumor RD, and age (Table 3). After multivariate analysis, which produced an HR of 1.698 (95% CI, 1.22–2.363; *p* = 0.002), along with primary therapy outcome, cancer status, and age, LYNX1 still independently associated with OS.

Other clinicopathological variables were associated with poor DSS including primary therapy outcome, cancer status, and tumor RD (Table 4). After multivariate analysis, which produced an HR of 1.566 (95% CI, 1.13–2.17; *p* = 0.007), along with primary therapy outcome, cancer status, and age, LYNX1 were still independently associated with DSS.

### 3.4. GSEA Identifies a LYNX1-Related Signaling Pathway

**Figure 3:**
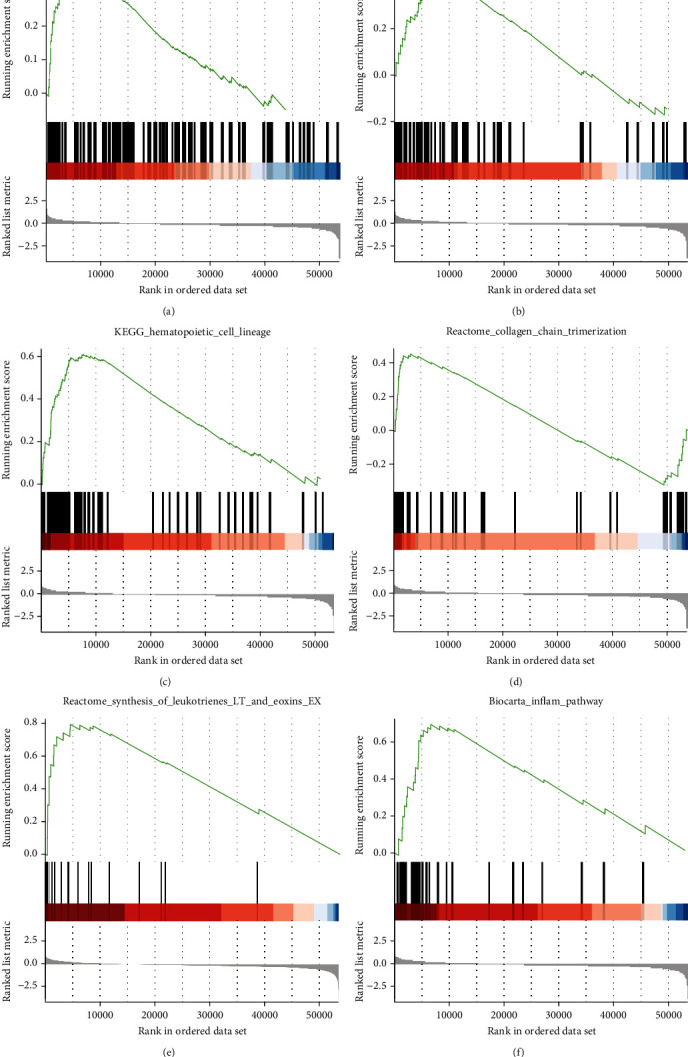
Enrichment plots from set enrichment analysis (GSEA). GSEA results showing MHC class II antigen (a), heparan sulfate (b), hematopoietic cell lineages (c), collagen chains (d), synthesis of leukotrienes (e), and inflammation pathway (f) are differentially enriched in LYNX1-related ovarian cancer.

To identify LYNX1-related enrichment signaling pathways in OVs, we used GSEA between low- and high-expression LYNX1. The expression of the gene in the ovarian cancer samples was divided into high-expression and low-expression LYNX1 with the median as the cut-off point. GSEA revealed significant differences (false discovery rate, FDR *q* value < 0.05; nominal, NOM *p* value < 0.05) in enrichment of the Molecular Signatures Database (MSigDB) Collection (c2.cp.reactome/biocarta/kegg.v6.2.symbols.gmt). We selected the most significantly enriched signaling pathways based on their NES (Figure 3 and Table 5). With high expression of LYNX1, this revealed differential enrichment of major histocompatibility complex (MHC) class II antigen, heparan sulfate, hematopoietic cell lineages, collagen chains, synthesis of leukotrienes, and inflammation pathway categories.

### 3.5. Correlation Analysis between LYNX1 Expression and Immune Cells

**Figure 4:**
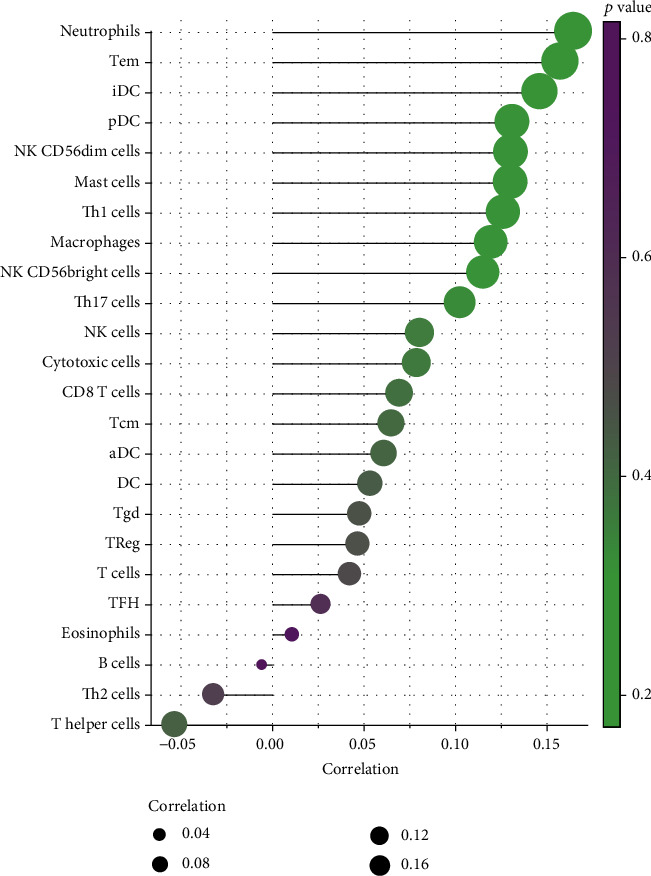
Correlation analysis between LYNX1 and related immune cells in ovarian serous cystadenocarcinoma (OVs).

We focused on the correlations between LYNX1 and immune cells of OVs in the databases of Bindea and colleagues to clarify their relation to each other [19]. This analysis revealed that LYNX1 expression is correlated with 24 types of immune cells. These include several T-cell subsets (e.g., effector memory (Tem), central memory (Tcm), *γδ*, regulatory (Treg), T helper (Th)1, Th2, Th17, T follicular helper, and cytotoxic T cells), three dendritic cell (DC) types (e.g., immature, activated, and plasmacytoid), and two subtypes of natural killer (NK) cells (CD56^dim^ and CD56^bright^), as well as neutrophils, mast cells, macrophages, eosinophils, and B cells (Figure 4).

The results revealed a strong positive relationship between LYNX1 and neutrophils. Moderately positive and significant correlations were found between lynx1 and Tem cells, immature dendritic cells (iDCs), plasmacytoid dendritic cells (pDCs), CD56^dim^ NK cells, mast cells, Th1 cells, Th17 cells, macrophages, and CD56^bright^ NK cells. A weak negative relationship arose between LYNX1 and the other T helper cell types (Figure 4).

## 4. Discussion

Lynx1 (Ly6/Neurotoxin 1) is a protein coding that encodes a GPI-anchored, cell-membrane-bound member of the Ly6/uPAR (LU) superfamily of proteins containing the unique three-finger LU domain [20]. This protein interacts with nAChRs and is thought to function as a modulator of nAChR activity to prevent excessive excitation. An important paralog of LYNX1 is Ly6D. The Ly6 family is known to coreside with c-myc on chromosome 8q24. In many cancer types, an increase in the somatic cell copy number of 8q is associated with the most common increase in copy number, but LYNX1 has not been extensively studied [21, 22]. In addition, high Ly6D expression is significantly correlated with poor clinical outcome in ovarian cancer [23]. Here, analysis results based on the high-throughput RNA sequencing data of TCGA showed that increased LYNX1 expression in OVs was associated with poor clinicopathological characteristics, shortened survival time, and poor prognosis. In addition, our analysis showed that LYNX1 expression levels in OVs were correlated with different types of immune-infiltrating cells. Therefore, our study provides evidence to support our understanding of the potential role of LYNX1 in OV immunity and as a potential diagnostic or prognostic marker of this malignancy.

In the present study, we used TCGA data to detect OVs prognosis and expression level of LYNX1. Previous studies have shown that LYNX1 mRNA and protein are expressed in lung, colon, epidermis, and breast cancer cells [14, 24]. Knockdown of LYNX1 increases growth of lung cancer cells by siRNAs, while enriched expression of LYNX1 in lung cancer cells decreases cell proliferation [14]. Based on these previous reports [14, 24], LYNX1 is a tumor suppressor in lung cancer, but it has been unclear whether LYNX1 is a tumor suppressor or an oncogenic in other cancers. Until now, there have been no studies into the role of LYNX1 in ovarian cancer.

Based on the TCGA database, we found that the OS and DSS rates of OVs patients decreased with the increase in LYNX1 expression. Univariate and multivariate regression analyses revealed that high LYNX1 expression correlates with poor OVs prognosis. Additionally, when LYNX1 was highly expressed in OVs, we found that higher levels of LYNX1 expression were correlated with poorer primary therapy and poorer cancer status for poorer OS and DSS. When put together, our findings suggest that LYNX1 is a prognostic biomarker for OVs.

Growing evidence demonstrates that ovarian cancer is essentially an immunogenic tumor. Epidemiological and clinical data show that survival of ovarian cancer patients is associated with spontaneous antitumor immune response and tumor immune escape mechanism [25]. Our current study found that LYNX1 expression is associated with a variety of immune infiltration cells. Neutrophils showed strong correlation with LYNX1 expression. Inflammation plays an important role in the development and progression of epithelial ovarian cancers, and a meta-analysis indicates that the preoperative neutrophil-to-lymphocyte ratio is an important predictor of prognosis in epithelial ovarian cancer patients [26]. Therefore, our findings reveal the potential regulating role of LYNX1 in inflammation with ovarian cancer. In addition, iDC, pDC, and aDC showed evident correlations with LYNX1 expression. DCs are classic antigen presenting cells. Immature DCs have a strong phagocytic ability, while mature DCs produce a large number of cytokines and have a strong regulation function [27]. These results showed that DCs had the potential to be activated by LYNX1. In addition, there was a significant correlation between the regulation of Tem cells and NK cells in OVs and LYNX1 expression. After response to target recognition, Tem cells and NK cells are activated and secrete interferon- (IFN-) *γ*, which has direct antiproliferative activity on ovarian cancer cells *in vitro* [28]. IFN-*γ* upregulates the human leukocyte antigen (HLA, also called MHC) class I and II molecules and antigen presentation in ovarian tumor cells *in vitro* and *in vivo* [29], a requisite for recognition by T cells. HLA class I expression by the tumor correlates with the intensity of T-cell infiltration [30], a predictor of longer survival. This correlation may suggest a potential mechanism by which LYNX1 regulates T-cell function in OVs. These findings therefore suggest that LYNX1 plays a crucial role in regulating immune cell infiltration and their inflammatory response in OVs.

It is unclear how LYNX1 expression is associated with immune infiltration and poor prognosis [14, 24]. LYNX1 may modulate *α*7-nAChR signaling in cancer cells [14] because LYNX1 is colocalized with *α*7-nAChRs in epithelial cells, and 90% of ovarian cancers are epithelial ovarian cancer.

The *α*7-nAChR protein is mainly distributed in the central and peripheral nervous systems but is also found in the lungs, muscles, and placenta. Signaling pathways associated with *α*7-nAChR are mainly concentrated in nicotine addiction, cancers, and preeclampsia [31, 32]. Macrophages in the brain show increased *α*7-nAChR expression, which inhibits the production of inflammatory cytokines [33]. In colorectal cancer, through the Janus kinase (JAK2)/signal transducer and activator of transcription (STAT3) signaling pathway, activation of *α*7-nAChR in tumor macrophages inhibits colorectal cancer metastasis [34]. The *α*7-nAChR has been recognized as an important drug target to inhibit lung cancer [35], and we speculate that it may also be an important drug target in epithelial ovarian cancer. Although the mechanism of nAChR signaling has not been reported for ovarian cancer, we speculate that LYNX1-related promotion of ovarian cancer cell growth may occur via modulation of *α*7-nAChR and activation of different intracellular signaling cascades.

In addition, many epithelial cells express a cholinergic autocrine loop in which Ach acts as a growth factor to stimulate cell growth. Cancers derived from these tissues similarly express a cholinergic autocrine loop, and Ach secreted by the cancer or neighboring cells interacts with M3 muscarinic receptors expressed on the cancer cells to stimulate tumor growth. Primary proliferative pathways involve mitogen-activated protein kinase (MAPK) and Akt activation. Fu et al. reported that the potential role of LYNX1 in modulating lung cancer cell growth is supported by the increase in cholinergic signaling reported in lung cancers in which levels of Ach and nicotinic receptors are increased [24, 36]. Ach is an essential neurotransmitter that regulates multiple functions of the female reproductive system. In physiological conditions, Ach regulates ovarian functions like ovarian hormone production [37] or growth and differentiation of ovarian follicles [38] and activates muscarinic receptors. Thus, LYNX1 may regulate the expression of *α*7- nAChR through different signaling pathways and change the levels of Ach and nicotinic receptors to regulate the growth of ovarian cancer cells.

This study has some limitations. First, the selected data setting samples were used only to distinguish whether or not they are tumor tissues without further classification of tumor stages. Second, we could not clearly evaluate the correlation between LYNX1 mRNA expression and its protein expression. A primary study [39] suggests that the use of mRNA expression to predict protein expression is not entirely accurate. Third, the function of this factor therefore needs further experimental verification, which requires crossvalidation of multiple data sets, coverification of *in vivo* and *in vitro* experiments, or coverification of multiple tumor sites. Finally, batch effects are almost inevitable. Batch effect means that some sources of variation are unrelated to inter- and intrasample class differences, and they arise from, for instance, different handlers, experiment times, instruments, and reagents [40]. These differences can confound biological variations of interest during data integration. To solve this problem, tools developed for microarray data batch correction such as ComBat [41] and limma [15] have been employed. According to our statistical conventions, we used the removeBatchEffect function in the limma package to correct batch effects. Nevertheless, the batch effect can only be weakened, not eliminated fundamentally.

## 5. Conclusions

In conclusion, increased LYNX1 expression predicted poor prognosis of OVs with increased infiltration of neutrophils and other immune cells. Moreover, LYNX1 expression in OVs potentially contributes to the regulation of neutrophils, memory T cells, NK cells, or DCs via modulation of *α*7-nAChRs and activation of different intracellular signaling cascades to alter the levels of Ach and nicotinic receptors related to the growth of OVs. Therefore, LYNX1 may play a crucial role in immune cell infiltration and as a prognosis biomarker in patients with OVs.

## Figures and Tables

**Table 1:** The Cancer Genome Atlas (TCGA) ovarian serous cystadenocarcinoma (OVs) patient characteristics.

Characteristics	Number of cases	Percentages
Clinical stage		
Stage I	1	0.27
Stage II	22	5.9
Stage III	293	78.55
Stage IV	57	15.28
Primary therapy outcome		
SD	22	7.28
PD	27	8.94
PR	43	14.24
CR	210	69.54
OS		
Alive	146	38.83
Dead	230	61.17
Cancer status		
Tumor free	85	25.68
With tumor	246	74.32
Histologic grade		
G1	1	0.27
G2	42	11.48
G3	322	87.98
G4	1	0.27
Tumor residual disease		
NRD	66	19.82
RD	267	80.18
Anatomic neoplasm subdivision		
Unilateral	101	28.53
Bilateral	253	71.47
Age		
<60	198	52.66
≥60	178	47.34

SD: stable disease; PD: progressive disease; PR: partial remission; CR: complete remission; OS: overall survival; NRD: no residual disease; RD: residual disease.

**Table 2:** LYNX1 expression associated with clinicopathological characteristics (logistic regression).

Characteristics	Total (*N*)	Odds ratio in LYNX1expression	*p* value
Clinical stage (stage I/II vs. stage III/IV)	373	0.92 (0.39–2.15)	0.84
Primary therapy outcome (SD-PD vs. PR-CR)	302	0.85 (0.46–1.57)	0.604
Cancer status (tumor free vs. with tumor)	331	1.22 (0.74–2)	0.433
Histologic grade (G1G2 vs. G3G4)	366	1.05 (0.56–2)	0.871
Tumor residual disease (NRD vs. RD)	333	2.08 (1.2–3.67)	0.01
Anatomic neoplasm subdivision (unilateral vs. bilateral)	354	0.82 (0.51–1.3)	0.391
Age (<60 vs. ≥60)	376	0.55 (0.36–0.82)	0.004

SD: stable disease; PD: progressive disease; PR: partial remission; CR: complete remission; NRD: no residual disease; RD: residual disease.

**Table 3:** Univariate and multivariate Cox proportional hazard analysis of LYNX1 expression and overall survival (OS) for patients with ovarian serous cystadenocarcinoma (OVs) in the validation cohort.

Characteristics	HR (95% CI) univariate analysis	*p* value univariate analysis	HR (95% CI) multivariate analysis	*p* value multivariate analysis
Clinical stage (stage I/II vs. stage III/IV)	2.085 (0.925–4.699)	0.076	2.328 (0.567–9.55)	0.241
Primary therapy outcome (SD-PD vs. PR-CR)	0.303 (0.205–0.447)	<0.001	0.336 (0.218–0.516)	<0.001
Cancer status (tumor free vs. with tumor)	8.466 (4.591–15.611)	<0.001	11.874 (4.798–29.383)	<0.001
Histologic grade (G1G2 vs. G3G4)	1.194 (0.797–1.789)	0.389		
Tumor residual disease (NRD vs. RD)	2.302 (1.479–3.583)	<0.001	1.094 (0.653–1.834)	0.734
Anatomic neoplasm subdivision (unilateral vs. bilateral)	1.041 (0.768–1.41)	0.798		
Age (<60 vs. ≥60)	1.329 (1.025–1.722)	0.032	1.384 (1.001–1.915)	0.049
LYNX1 (low vs. high)	1.414 (1.089–1.837)	0.009	1.698 (1.22–2.363)	0.002

SD: stable disease; PD: progressive disease; PR: partial remission; CR: complete remission; NRD: no residual disease; RD: residual disease; HR: hazard ratio; CI: confidence interval.

**Table 4:** Univariate and multivariate Cox proportional hazard analysis of LYNX1 expression and disease-specific survival (DSS) for patients with ovarian serous cystadenocarcinoma (OVs) in the validation cohort.

Characteristics	Univariate analysis		Multivariate analysis	
	HR (95% CI)	*p* value	HR (95% CI)	*p* value
Clinical stage (stage I/II vs. stage III/IV)	2.244 (0.922–5.462)	0.075	2.26 (0.551–9.271)	0.258
Primary therapy outcome (SD-PD vs. PR-CR)	0.295 (0.199–0.439)	<0.001	0.348 (0.227–0.535)	<0.001
Cancer status (tumor free vs. with tumor)	15.22 (6.731–34.413)	<0.001	19.489 (6.144–61.818)	<0.001
Histologic grade (G1G2 vs. G3G4)	1.313 (0.833–2.07)	0.24		
Tumor residual disease (NRD vs. RD)	2.559 (1.572–4.166)	<0.001	1.141 (0.671–1.938)	0.626
Anatomic neoplasm subdivision (unilateral vs. bilateral)	1.034 (0.747–1.431)	0.841		
Age (<60 vs. ≥60)	1.248 (0.944–1.65)	0.12		
LYNX1 (low vs. high)	1.393 (1.053–1.845)	0.02	1.566 (1.13–2.17)	0.007

SD: stable disease; PD: progressive disease; PR: partial remission; CR: complete remission; NRD: no residual disease; RD: residual disease; HR: hazard ratio; CI: confidence interval.

**Table 5:** Gene sets enriched in high phenotype.

Description	Set size	Enrichment score	NES	NOM *p* value	*p* adjust	FDR *q* values	Rank	Leading_edge
Reactome_synthesis_of_leukotrienes_LT_and_eoxins_EX	21	0.794	2.602	0.004	0.060	0.045	4526	Tags = 57%, list = 8%, signal = 52%
Biocarta_inflam_pathway	27	0.695	2.434	0.005	0.060	0.045	6632	Tags = 63%, list = 12%, signal = 55%
Reactome_heparan_sulfate_heparin_HS_GAG_metabolism	55	0.391	1.639	0.008	0.068	0.051	9176	Tags = 44%, list = 17%, signal = 36%
KEGG_hematopoietic_cell_lineage	84	0.609	2.813	0.010	0.069	0.051	7667	Tags = 61%, list = 14%, signal = 52%
Reactome_collagen_formation	90	0.467	2.138	0.012	0.071	0.053	3536	Tags = 36%, list = 7%, signal = 33%
Reactome_MHC_class_II_antigen_presentation	122	0.325	1.545	0.019	0.092	0.069	8284	Tags = 26%, list = 15%, signal = 22%

NES: normalized enrichment score; NOM: nominal; FDR: false discovery rate. Gene sets with NOM *p* value < 0.05 and FDR *q* value < 0.05 are considered as significant.

## Data Availability

The data used to support the findings of this study are available from the corresponding author upon request.
